# Psychosis screening questionnaire: Exploring its factor structure among South African adults

**DOI:** 10.4102/sajpsychiatry.v29i0.2051

**Published:** 2023-11-17

**Authors:** Yanga Thungana, Zukiswa Zingela, Stefan van Wyk, Hannah H. Kim, Amantia Ametaj, Anne Stevenson, Rocky E. Stroud, Dan J. Stein, Bizu Gelaye

**Affiliations:** 1 Department of Psychiatry, Faculty of Health, Walter Sisulu University, Mthatha, South Africa; 2 Faculty of Health, Nelson Mandela University, Port Elizabeth, South Africa; 3 Department of Social and Behavioral Sciences, Faculty of Public Health, Harvard T.H. Chan School of Public Health, Boston, United States of America; 4 Department of Epidemiology, Harvard T.H. Chan School of Public Health, Boston, United States of America; 5 Department of Psychiatry, Stanley Center for Psychiatric Research, Broad Institute of MIT and Harvard, Cambridge, United States of America; 6 Department of Psychiatry, Faculty of Health Sciences, Stellenbosch University, Cape Town, South Africa; 7 Department of Psychiatry and Mental Health, Faculty of Health Sciences, University of Cape Town, Cape Town, South Africa; 8 Division of Global Psychiatry, Massachusetts General Hospital and Harvard Medical School, Boston, United States of America

**Keywords:** psychosis, assessment, psychosis screening questionnaire, South Africa, early detection

## Abstract

**Background:**

Early detection of psychosis improves treatment outcomes, but there is limited research evaluating the validity of psychosis screening instruments, particularly in low-resourced countries.

**Aim:**

This study aims to assess the construct validity and psychometric properties of the psychosis screening questionnaire (PSQ) in South Africa.

**Setting:**

This study was conducted at several health centres in the Western and Eastern Cape provinces in South Africa.

**Methods:**

The sample consisted of 2591 South African adults participating as controls in a multi-country case-control study of psychiatric genetics. Using confirmatory factor analysis and item response theory, we evaluated the psychometric properties of the PSQ.

**Results:**

Approximately 11% of the participants endorsed at least one psychotic experience on the PSQ, and almost half of them (49%) occurred within the last 12 months. A unidimensional model demonstrated good fit (root mean square error of approximation [RMSEA] = 0.023, comparative fit index [CFI] = 0.977 and Tucker–Lewis Index [TLI] = 0.954). The mania item had the weakest association with a single latent factor (standardised factor loading = 0.14). Model fit improved after removing the mania item (RMSEA = 0.025, CFI = 0.991 and TLI = 0.972). With item response theory analysis, the PSQ provided more information at higher latent trait levels.

**Conclusion:**

Consistent with prior literature, the PSQ demonstrated a unidimensional factor structure among South Africans. In our study, the PSQ in screening for psychosis performed better without the mania item, but future criterion validity studies are warranted.

**Contribution:**

This study highlights that PSQ can be used to screen for early psychosis.

## Introduction

The lifetime prevalence of psychotic disorders is estimated to be 1% to 3% worldwide.^1,2^ Despite the low prevalence, psychotic disorders like schizophrenia are among the world’s leading causes of disability and morbidity.^3,4,5^ Notably, people diagnosed with psychosis are more likely to die around 10 years earlier than the general population.^6,7,8^ In contrast to psychotic disorder, psychotic-like experiences (PLE) are much more common in the general population.^9,10^ Psychotic-like experiences are transient for most people, but they can be a harbinger of future psychotic disorders.^11,12^ Moreover, those experiencing PLEs are at increased risk for developing other psychiatric disorders such as anxiety, mood and substance use disorders.^13,14,15^ Hence, PLEs may reflect an underlying susceptibility to a broad range of negative mental health outcomes, highlighting the importance of early detection of PLEs.

The duration of untreated psychosis is associated with unfavourable outcomes, including frequent hospitalisation, inadequate response to treatment and limited functional recovery.^16,17,18,19,20^ Early detection and shorter duration of untreated psychosis improve the treatment outcomes of patients with psychotic illness.^17,18^ Screening tools for many psychiatric disorders, including psychotic disorders, aid in early diagnosis, which, in turn, may be associated with a better prognosis.^21^ Unfortunately, existing clinician-administered tools used to detect the presence of psychotic features^22^ are often not suitable for routine clinical practice and population-based epidemiologic surveys. These measures are typically lengthy and require specialised training.^21^ Thus, screening tools that are practical and easy to administer without the need for clinical training may aid in the early diagnosis and reductions in disability and morbidity from psychotic disorders.

In low- and middle-income countries such as South Africa, screening tools administered by laypersons may be particularly beneficial given the limited mental health workforce.^23,24,25,26^ Unfortunately, there are no clinician-administered screening tools for psychosis that have been validated in South Africa, layperson- or clinician-administered. To the best of our knowledge, the only South African study was of a self-report psychosis screening tool (Community Assessment of Psychic Experiences), which performed poorly in screening for psychosis.^27^

To close the gap in research on screening tools for psychosis in South Africa, we examined the psychometric properties of the psychosis screening questionnaire (PSQ).^28^ The PSQ is a self-reported measure that has been studied primarily in Western settings.^29,30,31^ However, to the best of our knowledge, no validation studies of the PSQ as a screening tool for psychosis have been done in the South African population. There is a published study on the cross-cultural examination of the PSQ across Uganda, Ethiopia, Kenya and South Africa.^32^ However, this study was focused on a broad comparison of the scales’ performance across the four countries to test its equivalence across settings without including the specifics of the PSQ’s performance from each country. Our study is focused on the PSQ as used in South Africa and will examine the measure performance in detail in our setting, including item-level data with item response theory (IRT).

In this study, we sought to evaluate the construct validity of the PSQ (i.e. factor structure) using data collected from a large South African sample, which is part of a more extensive epidemiological research study on the genetics and phenotypic symptoms of neuropsychiatric disorders across four African countries.^33^ We also sought to better understand the latent construct of the PSQ in the South African context using IRT analytic approaches. Item response theory models allow for a better understanding of how the PSQ performs in a specific population. The models relate characteristics of items and attributes of individuals to the probability of selecting various responses of an item on a scale.

## Research methods and design

The Neuropsychiatric Genetics of African Population-Psychosis Study (NeuroGAP-Psychosis) is a case-control, genome-wide association study (GWAS) aiming to advance the understanding of genetic and environmental risk factors of psychotic disorders in Africa, including Ethiopia, Kenya, South Africa and Uganda.^33^ Data for the current research project are based on participants from South Africa.

### Participants

NeuroGAP-Psychosis participants were recruited commencing in April 2018, and the analysis for this study is limited to data from South Africa through March 2020. In South Africa, the controls, who are the focus of this study, were enrolled from a large academic hospital in the Eastern Cape, a psychiatric hospital and various community clinics in the Western Cape. Individuals who were controls did not have a clinical diagnosis of psychosis (schizophrenia and bipolar disorder) and sought general medical care, students or staff at the facilities or family members of those seeking care. Ethical clearance to conduct the study was obtained from all the participating sites, including the Research Ethics Committees of the two universities involved, the Western Cape Department of Health and the Eastern Cape Department of Health. Approval was also obtained from the Harvard T.H. Chan School of Public Health IRB in the United States.

### Demographics

Demographic details such as age, sex, marital status, participant’s preferred language, living circumstances and level of education that were used during analysis to characterise the sample further was also collected.

### Psychosis screening questionnaire

The PSQ is a screening tool designed to detect self-reported psychotic symptoms in the general population.^28^ The measure has five root questions that assess the presence of PLE (mania, thought insertion, paranoia, strange experiences and perceptual disturbances).^29,34^ Each root question is followed by one or two additional questions to collaborate on such occurrence as being symptomatic of psychosis. A dichotomous measure (present or absent) for each of the five symptoms was derived. The screening test for psychosis was considered positive if a person responded affirmatively to any of the five root questions and their corresponding targeting questions.^28^ Furthermore, the positive results were categorised into past-year and lifetime occurrences.

### Data analytic plan

The characteristics of the study population was first examined using means and standard deviations for continuous variables and using counts and percentages for categorical variables. Next, the prevalence of psychotic symptoms in the study population was calculated.

#### Confirmatory factor analysis

A confirmatory factor analysis (CFA) of the PSQ was conducted. In addition, a unidimensional factor structure was examined based on prior literature.^28,31,35^ To the best of our knowledge, one previous study examined the factor structure of PSQ for a British sample of multiple ethnic groups and found a unidimensional factor structure to best fit the data.^31^ A traditional split sample exploratory-CFA was not conducted because of a floor effect in the data owing to the low prevalence of psychotic disorders in the study population. Confirmatory factor analysis was performed in Mplus 8 v.1.7.^36^

Model fit was evaluated with the following metrics: (1) root mean square error of approximation (RMSEA) defined as 0.060 or below for a well-fitting model^37^; (2) comparative fit index (CFI) with good fit indicated by 0.90 or above^37,38^ and (3) Tucker–Lewis Index (TLI) with a good fit of close to 0.90 or above.^37^

#### Item response theory

Item response theory analyses were conducted *via* the following steps: Firstly the three assumptions required for an IRT model, namely, unidimensionality, local independence and monotonicity, was tested. To test unidimensionality, the fit of the data to a one-factor CFA model was investigated. Secondly, the matrix of the residual correlations from the one-factor CFA was examined to test local independence. Finally, monotonicity plots were visually assessed using Mokken scaling. After checking the assumptions, a unidimensional latent structure, 2-parameters logistic model was fit. This model accounts for the difficulty of implementing each functionality (i.e. how well items identify individuals at different levels of the latent trait) and discrimination (i.e. the rate at which the probability of endorsing the item changes given the latent trait) of each PSQ item. Item information curves (IIC), item characteristic curves and the total information curves were generated using the R statistical program, version 3.6.2, packages *Mokken* and *ltm*.

*Item difficulty (bi)* is the parameter that determines how the item behaves along the latent trait scale. When examining discrimination parameters, we chose to focus on items that peak at high levels of θ, approximately 2–4 standard deviations above the mean, which represent moderate to high levels of psychosis. *Item discrimination (ai)* refers to the degree to which an item discriminates between individuals with different levels of the latent trait (i.e. psychosis). In other words, it is the probability of endorsing a PSQ item given the underlying psychosis levels.

### Ethical considerations

Ethical approval to conduct this study was obtained from all participating sites, including the University of Cape Town Human Research Ethics Committee (REF# 466/2016), the Western Cape Government (WC_2016RP32_349) and the Walter Sisulu University Research and Ethics Committee (SOMREC #REC REF 2016-057) in South Africa and the Harvard T.H. Chan School of Public Health (#IRB17-0822) in the United States. All experimental protocols were approved by the above-mentioned institutions and/or ethics committees. Informed consent was obtained from all study participants, and all experiments were conducted in accordance with the relevant guidelines and regulations.

## Results

The characteristics of the study participants are summarised in Table 1. The final analytic sample consisted of 2591 participants. The mean age of the participants was 35 years (standard deviation = 11.7) with slightly more female participants (51.6%). Most of the study participants were single (55.4%) and had secondary education (72.4%). Differences in living arrangements and additional details on demographic information for the sample are depicted in Table 1.

**TABLE 1:** Participant demographics of South Africa (*N* = 2591).*

Variable	Count	%
**Sex**		
Female	1337	51.6
Male	1254	48.4
**Age categories†**		
18–35	1467	56.6
35–59	1037	40.0
60+	87	3.4
**Marital status**		
Single	1436	55.4
Married or cohabitating	880	34.0
Widowed	64	2.5
Divorced or separated	204	7.9
**Level of education**		
No formal	8	0.3
Primary	218	8.4
Secondary	1877	72.4
University	486	18.8
**Living arrangements**		
Lives alone	610	23.6
Lives with parental family	630	24.3
Lives with spouse or partner	875	33.8
Lives with friends or other relatives	453	17.5
Unknown or missing	23	0.8

* , Counts may not add up to the total because of missing information for some participants.

† , mean = 35.4 s.d. = 11.7

**FIGURE 1:**
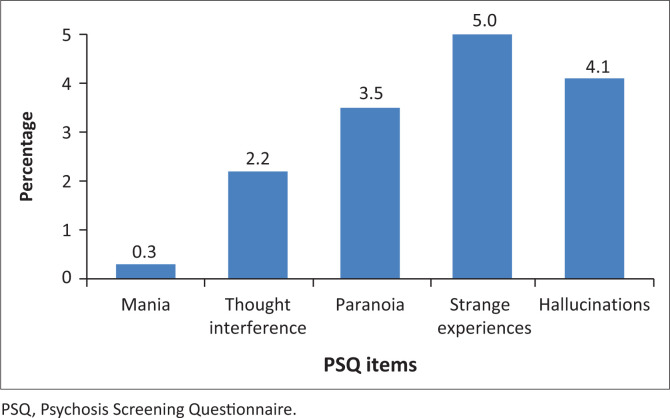
Prevalence of positive screen items on psychosis screening questionnaire in South Africa (*n* = 2591).

Next, we examined the prevalence of psychotic symptoms (Figure 1). Approximately 11% of the study participants reported psychotic experiences, and of those, 49.1% of them experienced psychotic symptoms within the last 12 months. The prevalence of strange experiences was the highest (5.0%), followed by hallucinations (4.1%), paranoia (3.5%) and thought interference (2.2%). Mania was the least endorsed symptom. The prevalence of psychotic experiences was equally distributed among female participants (*n* = 154; 52.6%) and male participants (*n* = 139; 47.4%). Prevalence was highest among the middle-aged (50.5%), followed by young adults (45.1%) and older adults (4.4%). The participants in the study used one of the three languages, namely, English (49.1%), Xhosa (44.8%) and Afrikaans (6.1%). The proportion of psychotic experiences varied between people speaking Xhosa, English and Afrikaans as follows: hallucinations (49.5%, 38.1%, 12.4%), paranoia (54.95%, 42.86%, 2.2%), thought interference (55.2%, 54.8%, 0.0%), strange experiences (46.1%, 50.4%, 3.1%) and mania (0.0%, 87.5%, 12.5%), respectively.

The authors conducted a CFA using the unidimensional factor structure to examine the fit and parameter statistics of the PSQ (see Table 2a and Table 2b). The unidimensional model provided a good fit for the data (RMSEA = 0.023; CFI = 0.977; TLI = 0.954), but the mania item showed only a weak association with the underlying latent factor (standardised factor loading [s.e.] = 0.14). Thus, we re-ran the factor analysis without the mania item and observed an improvement in the fit of the model (RMSEA = 0.025; CFI = 0.991; TLI = 0.972). In addition, Table 2 shows the unidimensional model of psychosis for the PSQ in South Africa with strong factor loadings ranging from 0.69 to 0.79 without the mania item.

**TABLE 2a:** Model fit and parameter estimates for confirmatory factor analysis of psychosis screening questionnaire in South Africa sample with and without mania items (*N* = 2591).

Variable	Fit statistic						
	χ^2^	df	*p*	RMSEA	90% CI	CFI	TLI
1-factor solution with the mania item	15.98	8	0.043	0.023	0.006 to 0.041	0.977	0.954
1-factor solution without the mania item	10.31	4	0.036	0.025	0.000 to 0.052	0.991	0.972

RMSEA, root mean square error of approximation; CFI, comparative fit index; TLI, Tucker–Lewis Index

**TABLE 2b:** Model fit and parameter estimates for confirmatory factor analysis of psychosis screening questionnaire in South Africa sample with and without mania items (*N* = 2591).

PSQ item	Model results			
	Standardised factor loadings	s.e.	Standardised factor loadings	s.e.
Thought interference	0.71	0.06	0.69	0.06
Paranoia	0.62	0.06	0.61	0.06
Strange experience	0.78	0.05	0.79	0.05
Hallucination	0.75	0.05	0.74	0.05
Mania	0.14	0.07	-	-

PSQ, Psychosis Screening Questionnaire, s.e. standard error

### Item response theory

**FIGURE 2:**
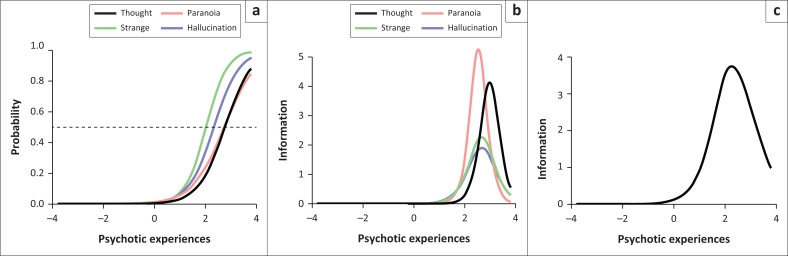
Item response theory – (a) Item characteristic curves, (b) Item information curves and (c) Test information function.

We decided to drop the mania item for the final IRT analysis because the monotonicity assumption was violated when mania was included in the model. Without the mania item, the monotonicity assumption was satisfied. As shown in the item characteristics curve (ICC; Figure 2a), strange experiences were easiest to endorse (farthest on the left). At the same time, thought disturbance and paranoia were the most difficult items to endorse. Strange experiences had the steepest slope suggesting it has the highest discriminability. The figure also demonstrates that the items – paranoia and thought abnormalities – have similar discrimination and, therefore, may convey similar information. The IIC graph indicated that the thought abnormalities item provided the most information at high latent levels. In contrast, paranoia, strange experiences and hallucinations provided more information at somewhat lower trait levels. Finally, the test information function (Figure 2c), the sum of the individual IICs, indicated that the PSQ provided information only at higher trait levels.

## Discussion

In this study, we examined the psychometric properties of the PSQ in a large South African sample of controls who did not have a clinical diagnosis of psychosis. The overall lifetime prevalence of psychotic symptoms was 11%, with strange experiences (5%) as the most prevalent psychotic symptom while mania (0.3%) was the least endorsed. The results of the CFA-confirmed items on the PSQ likely comprise one latent factor based on the CFI (0.977) and root mean square error value (0.023). However, the mania item showed a weak association with the underlying latent trait, psychosis. The IRT analysis showed that the PSQ provided high information only at higher levels of the underlying construct, which indicates that the PSQ will help identify individuals with a high level of psychosis compared to individuals with a low level of psychosis, further supporting the construct validity of the PSQ in South Africa.

The findings on prevalence estimates were difficult to compare to prior research because, in South Africa, there is a lack of reliable incidence data on psychotic disorders. In general, the prevalence of psychotic disorders is relatively low at about 1% – 3%^1,2^, and sub-Saharan Africa may have even lower rates of psychotic disorders.^39,40,41^ However, PLEs are much more common in the general population than psychotic disorders.^9,10^ The prevalence of PLEs varies significantly between countries; for instance, estimates range from as low as 0.8% to as high as 31.4%.^42^ There is some evidence from extensive comparative country studies showing that in some African communities, there tends to be a higher prevalence of PLEs.^43,44^ However, other large studies have failed to find a higher prevalence of PLEs in African countries.^42,45^ But several other studies conducted in different African countries have found a higher prevalence of PLEs in African communities.^42,46,47,48^ However, most of these studies were conducted in adolescents and young adults, a group associated with higher rates of PLEs.^49,50^ In our study, about 11% of participants had PLEs, which is on the high end compared to many Western studies, but lower than the previously documented South African prevalence of 16% described in a large cross-national study.^42^ The notable variation in PLEs between studies could be because of the difference in the age of study participants, the content of the scales used, the model of data collection (self-report vs. interviewer-administered) and inherent differences across populations.^51,52^ Additionally, culture plays a vital role in the experience, understanding and labeling of PLE.^53,54,55^

In our study, the endorsement of psychotic symptoms varied depending on the participant’s language; for instance, Xhosa-speaking participants had the highest prevalence of hallucinatory experiences. Of note, the primary language in South Africa often represents race and ethnicity. There is some evidence showing that performance on the individual items of the PSQ varies between ethnic groups.^31^ Also, there is evidence that the content and associated distress of the psychotic symptoms are influenced by the individual’s culture and the society they live in.^55,56^ Hence, it may not be surprising to find higher rates of perceptual disturbances among Xhosa-speaking people considering that interacting with ancestors, including receiving messages from them, is an acceptable practice in their culture. Furthermore, the language used to interview participants may influence the results of the screening tests; for instance, evidence shows that people not interviewed in their primary language may be more likely to endorse psychotic features with the PSQ.^34^ To counteract these language-related effects, all participants in our study were interviewed in their primary language.

The PSQ performed well as a unidimensional construct on the confirmatory analysis. Our study provides further evidence for the weak association of the mania item with the latent trait.^31^ This is not surprising considering that typically with mania, psychosis occurs in the background of a mood disturbance, and it usually consists of grandiose delusions and disordered speech. This contrasts with the odd ideations, thought disorder and paranoia captured by the PSQ items. Additional studies of this nature are needed to confirm our findings, specifically to evaluate the suitability and possible amendment of the mania item on the PSQ scale, especially in the African context.

The CFA and IRT showed that items assessing strange experiences and hallucinations gave the most precise information regarding psychosis as a measured latent trait compared to other items. The perception of the strangeness of experiences may differ between societies cross-culturally. For example, in non-Western countries, people might be more likely to endorse experiences such as feeling the presence of supernatural forces or communicating with the deceased because such experiences may have a higher value and cultural meaning in these communities, which can easily be recorded as strange on the screening scales.^32,57,58,59^ However, as shown in our IRT analysis, the PSQ provides useful information about the psychosis construct at higher levels of the latent trait, which should facilitate detecting mainly the clinical levels of psychosis.

### Limitations

The large sample size in an understudied population and the use of rigorous analytic techniques highlight some of the strengths of this study. However, some limitations should be considered when interpreting the results of our research. Firstly, our study did not utilise a clinical diagnostic gold or reference standard to assess criterion validity. Secondly, psychotic experiences were low prevalent, which did not allow evaluating measurement invariance analysis by key demographic and clinical characteristics. Lastly, the study recruited only participants attending general hospital healthcare settings. Hence, the findings may not be generalised to other populations.

## Conclusion

To the best of our knowledge, this is the first study to assess the psychometric properties of the PSQ in South Africa. Our findings suggest good construct validity and a one-dimensional structure for the PSQ in South Africa with a non-clinical population. In addition, using the PSQ to screen for psychosis may be better without the mania item. Future studies that examine the criterion validity of the PSQ are warranted.
